# Prognostic and clinical impact of PIK3CA mutation in gastric cancer: pyrosequencing technology and literature review

**DOI:** 10.1186/s12885-016-2422-y

**Published:** 2016-07-07

**Authors:** Kazuto Harada, Yoshifumi Baba, Hironobu Shigaki, Takatsugu Ishimoto, Keisuke Miyake, Keisuke Kosumi, Ryuma Tokunaga, Daisuke Izumi, Mayuko Ohuchi, Kenichi Nakamura, Yuki Kiyozumi, Junji Kurashige, Masaaki Iwatsuki, Yuji Miyamoto, Yasuo Sakamoto, Naoya Yoshida, Masayuki Watanabe, Hideo Baba

**Affiliations:** Department of Gastroenterological Surgery, Graduate School of Medical Science, Kumamoto University, 1-1-1 Honjo, Kumamoto, 860-8556 Japan; Department of Gastroenterological Surgery, Cancer Institute Hospital, Japanese Foundation for Cancer Research, Tokyo, Japan

**Keywords:** PIK3CA mutation, Gastric cancer, Pyrosequencing, Prognosis

## Abstract

**Background:**

Phosphatidylinositol-4,5-bisphosphate 3-kinase, catalytic subunit alpha (PIK3CA) mutations that activate the PI3K/AKT signaling pathway have been observed in several types of carcinoma and have been associated with patient prognosis. However, the significance of PIK3CA mutations in gastric cancer remains unclear. This retrospective study investigated the relationship between PIK3CA mutations and clinical outcomes in patients with gastric cancer. Additionally, we reviewed the rate of PIK3CA mutations in gastric cancer and the association between PIK3CA mutations and prognosis in human cancers.

**Methods:**

The study included 208 patients with gastric cancer who underwent surgical resection at Kumamoto University Hospital, Japan, between January 2001 and August 2010. Mutations in PIK3CA exons 9 and 20 were quantified by pyrosequencing assays.

**Results:**

PIK3CA mutations were detected in 25 (12 %) of the 208 patients. Ten patients had c.1634A > G (p.E545G), 10 had c.1624G > A (p.E542K), 13 had c.1633G > A (p.E545K), nine had c.3139C > T (p.H1047R), and 1 had c.3140A > G (p.H1047Y) mutations. PIK3CA mutations were not significantly associated with any clinical, epidemiologic, or pathologic characteristic. Kaplan–Meier analysis showed no significant differences in disease-free survival (log rank *P* = 0.84) and overall survival (log rank *P* = 0.74) between patients with and without PIK3CA mutations.

**Conclusions:**

Mutations in PIK3CA did not correlate with prognosis in patients with gastric cancer, providing additional evidence for the lack of relationship between the two.

## Background

Gastric cancer is the third leading cause of cancer deaths in the world, with 723,000 patients dying of gastric cancer in 2012 [1]. Elucidating the biological pathways leading to the development of gastric carcinoma is crucial because accumulation of genetic alterations has been shown to result in tumor development [2]. Genetic alterations involving proteins along several signaling pathways, such as the constitutive activation of receptor tyrosine kinases and G-protein-coupled receptors, and GTP-binding proteins to adaptor proteins, could lead to activation of the phosphoinositide 3-kinase (PI3K)-AKT pathway [3–6]. As PI3K signaling plays essential roles in cell growth, metabolism, survival, metastasis, and resistance to chemotherapy, the PI3K-AKT pathway has been considered extremely important in the carcinogenic process [7–10].

Mutations in the phosphatidylinositol-4,5-bisphosphate 3-kinase, catalytic subunit alpha (PIK3CA) gene, which encodes the p110 catalytic subunit of PI3K, have been found in several types of carcinoma [11, 12]. The hot spots of PIK3CA mutations have been located at five sites in exons 9 and 20 [13]. These mutations activate the PI3K/AKT signaling pathway, activating downstream signaling pathways, and thereby contribute to carcinogenesis [13]. PIK3CA mutations in several types of human cancer have been associated with patient prognosis. However the impact of PIK3CA mutations on prognosis varies among tumor types [14].

The significance of PIK3CA mutations in gastric cancer remains unclear. Although several reports have shown a relationship between PIK3CA mutations and prognosis, relative few patients were analyzed and findings differ among studies. This study used pyrosequencing to evaluate PIK3CA mutations in patients with gastric cancer at our hospital, as well as determining the relationship between PIK3CA mutations and patient prognosis. Additionally, we reviewed the rate of PIK3CA mutations in gastric cancer and the association between PIK3CA mutations and prognosis in human cancers.

## Methods

### Study subjects

This study retrospectively enrolled 208 gastric cancer patients who underwent resection at Kumamoto University Hospital between January 2001 and August 2010. Patients who underwent palliative resection and/or whose tissue samples were unavailable were excluded, but patients positive on peritoneal washing cytology were included. Patients were followed-up at 1- to 3-month intervals until death or 31 March 2015, whichever came first. Disease-free survival was defined as the time from the date of surgery to the date of cancer recurrence or death. Tumors were staged according to the American Joint Committee on Cancer Staging Manual (7th edition) [15]. Overall survival was defined as the time from the date of the operation to the date of death. All procedures followed were in accordance with the ethical standards of the responsible committee on human experimentation (institutional and national) and with the Helsinki Declaration of 1964 and later versions. Informed consent or substitute for it was obtained from all patients for being included in the study. The study procedures were approved by the Ethics Committee for Epidemiological and General Research at the Faculty of life Science, Kumamoto University (Approval number: Ethic 559). Throughout this article, the definition of “prognostic marker” is consistent with REMARK Guidelines [16].

### Genomic DNA extraction

Genomic DNA was extracted from paraffin-embedded tissue specimens of surgically resected gastric cancers. Tumors areas were marked on hematoxylin and eosin stained slides by one pathologist (Y. Baba). Genomic DNA was extracted from tumor lesions enriched with neoplastic cells, without adjacent normal tissue, using FFPE Kits (Qiagen, Duesseldorf, Germany).

### Pyrosequencing for PIK3CA mutations

**Fig. 1:**
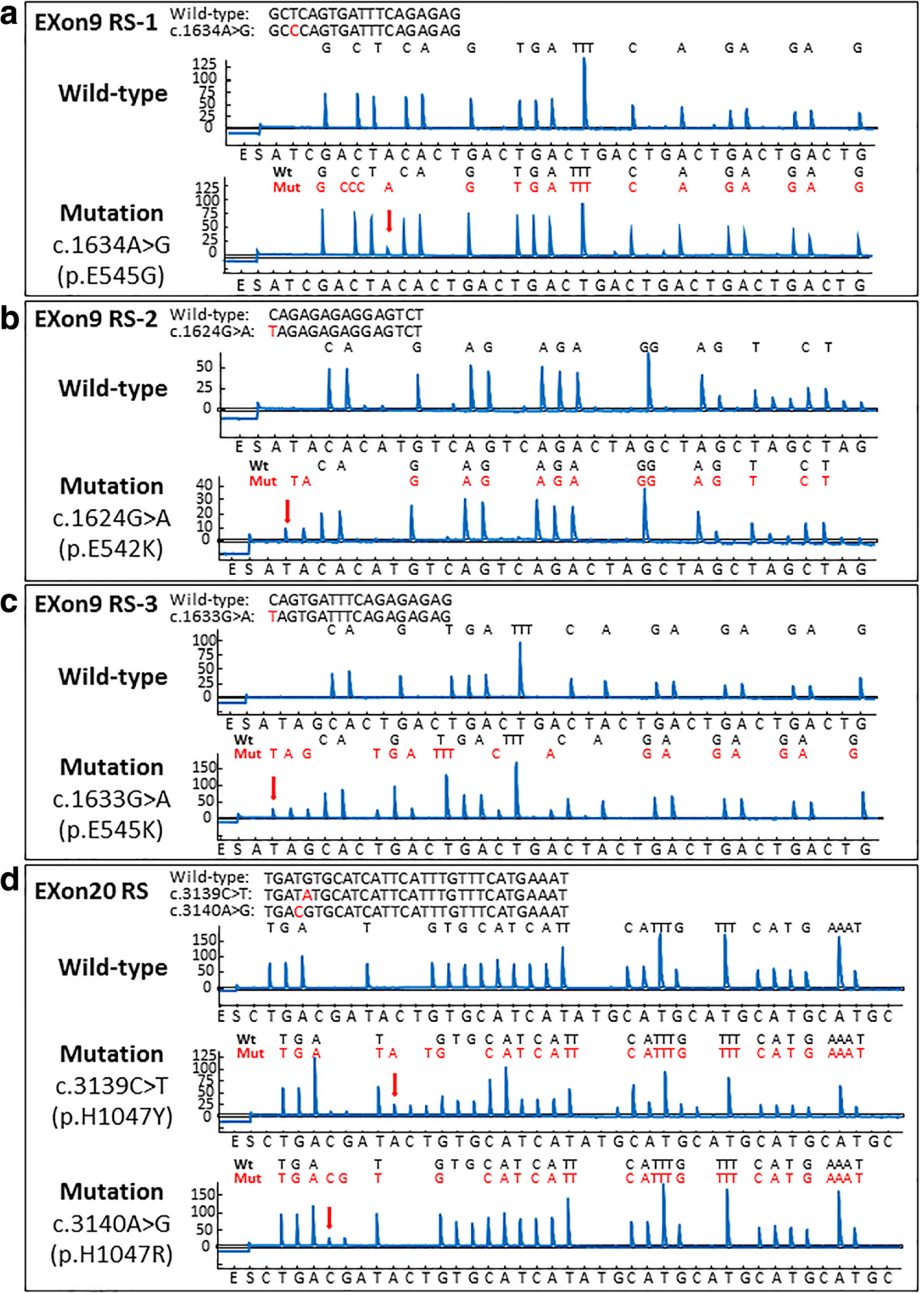
PIK3CA exon 9 and exon 20 pyrograms (antisense strand). **a** Wild-type exon 9 sequenced with the 9-RS1 primer and the c.1634A > G mutation (*arrow*), causing a shift in the reading frame and a new peak at A (*arrowhead*), which serves as quality assurance. **b** Wild-type exon 9 sequenced with the 9-RS2 primer and the c.1624G > A mutation (*arrow*), causing a shift in the reading frame and a new peak at A (*arrowhead*). **c** Wild-type exon 9 sequenced with the 9-RS3 primer and the c.1633G > A mutation (*arrow*), causing a shift in the reading frame and new peaks (*arrowheads*). **d** Wild-type exon 20 sequenced with the exon 20 primer and the c.3139C > T mutation (*arrow*), causing a shift in the reading frame and a new peak at T (*arrowhead*). Wild-type exon 20 sequenced with the exon 20 primer and the c.3140A > G mutation (*arrow*), causing a shift in the reading frame and a new peak at G (*arrowhead*). Mut, mutant; WT, wild-type

PIK3CA mutations were evaluated as previously described [14]. The exon 9 PCR primers were 5′-biotin-AACAGCTCAAAGCAATTTCTACACG-3′ (forward, PIK3CA 9-F) and 5′-ACCTGTGACTCCATAGAAAATCTTT-3′ (reverse, PIK3CA9-R). The exon 20 PCR primers were 5′-biotin-CAAGAGGCTTTGGAGTATTTCA-3′ (forward, PIK3CA 20-F) and 5′-CAATCCATTTTTGTTGTCCA-3′ (reverse, PIK3CA 20-R). In the PIK3CA exon 9 pyrosequencing assays, the presence of mutations was routinely confirmed using three different sequencing primers and by the creation of a frameshifted reading of a mutant sequence relative to a wild-type sequence. The primers PIK3CA 9-RS1 (5′-CCATAGAAAATCTTTCTCCT-3′; nucleotide dispensation order, ATCGACTACACTGACTGACTGACTGACTGACTGACTG), PIK3CA 9-RS2 (5′ TTCTCCTTGCTTCAGTGATTT-3′; nucleotide dispensation order, ATACACATGTCAGTCAGACTAGCTAGCTAGCTAG) and PIK3CA 9-RS3 (5′-TAGAAAATCTTTCTCCTGCT-3′; nucleotide dispensation order, ATAGCACTGACTGACTGACTACTGACTGACTGACTG) could detectc.1634A > G, c.1624A > G, and c.1624G > A mutations, respectively. For PIK3CA exon 20, the primer PIK3CA 20-RS (5′-GTTGTCCAGCCACCA-3′; nucleotide dispensation order, CTGACGATACTGTGCATCATATGCATGCATGCATGCATGC) was used to detect the mutations c.3140A > G and c.3139C > T. Nucleotide dispensation orders were designed so that if any of the common mutations were present, it caused a shift in the reading frame and resulted in an additional peak or peaks following the mutated nucleotide. Representative pyrograms of wild-type and mutant exons 9 and 20 are shown in Fig. 1.

### Statistical methods

The results were statistically analyzed by JMP software (Version 9, SAS Institute, Cary, NC, USA). All p-values were two-sided. The means were compared by *t* test, assuming unequal variances. The proportional differences among the characteristics were evaluated by Pearson’s chi-squared test (*χ*^2^). Survival time was determined by the Kaplan–Meier method and compared using the log-rank test. The hazard ratio for PIK3CA mutations on mortality was assessed by Cox regression modeling. To assess whether any of these variables was associated with the relationship between the PIK3CA mutations and prognosis, they were cross-multiplied with PIK3CA mutations and subjected to the Wald test.

## Results

### PIK3CA mutational status in gastric cancer

Of the 208 patients who had undergone curative resection of gastric cancer, 25 (12 %) were found pyrosequencing technology to have PIK3CA exon 9 and 20 mutations (Fig. 1). Ten patients were positive for c.1634A > G (p.E545G), 10 for c.1624G > A (p.E542K), 13 for c. c.1633G > A (p.E545K), nine for c. 3139C > T (p.H1047R), and one for c. 3140A > G (p.H1047Y) mutations. Three tumors were positive for mutations at all three nucleotides in exon 9, three were positive for mutations at two nucleotides in exon 9. Fifteen samples were positive for mutations in exon 9 alone, four for mutations in exon 20 alone, and six were positive for mutations in both exons.

### PIK3CA mutations and patient characteristics

PIK3CA mutations were not significantly associated with any clinical, epidemiologic, or pathologic characteristic (Table 1). We previously reported that the long interspersed nucleotide element-1 (LINE-1) methylation level in gastric cancer was a surrogate marker of global DNA methylation and genome instability [17, 18]. However, LINE-1 methylation level was not associated with PIK3CA mutations (Table 1).

**Table 1:** PIK3CA mutational status and clinical features in gastric cancers

Features	Total	PIK3CA mutation		*p* value
		Mutant	Wild-Type	
	*n* = 208	*n* = 25 (12 %)	*n* = 183 (88 %)	
Age (year)				0.30
Mean ± SE	208	67.4 ± 2.1	69.8 ± 0.8	
Sex				0.41
Male	148	16 (11)	132 (89)	
Female	60	9 (15)	51 (85)	
Location				0.22
Upper	75	13 (17)	62 (83)	
Middle	65	6 (9)	59 (91)	
Lower	68	6 (9)	62 (91)	
Tumor depth				0.09
T1	105	18 (17)	87 (83)	
T2	29	2 (6)	29 (94)	
T3	42	2 (5)	42 (95)	
T4	25	3 (11)	25 (89)	
Lymph node involvement				0.73
Absent	135	17 (13)	118 (87)	
Present	73	8 (11)	65 (83)	
Stage				0.78
I	123	17 (14)	106 (86)	
II	40	4 (10)	36 (90)	
III	26	2 (8)	24 (92)	
IV	19	2 (11)	17 (89)	
Lauren classification				0.79
Intestinal	138	16 (12)	122 (88)	
Diffuse	70	9 (13)	61 (87)	
Venous invasion				0.05
Absent	111	18 (16)	93 (84)	
Present	95	7 (7)	88 (93)	
Lymphatic invasion				0.40
Absent	117	16 (14)	101 (86)	
Present	91	9 (10)	82 (90)	
LINE-1 methylation level (%)				0.19
Mean ± SE	208	74.9 ± 2.6	71.2 ± 1.0	

*PIK3CA* phosphatidylinositol-4,5-bisphosphate 3-kinase catalytic subunit alpha, *LINE-1* long interspersed nucleotide element-1, *SE* standard error

### PIK3CA mutations and patient survival

**Fig. 2:**
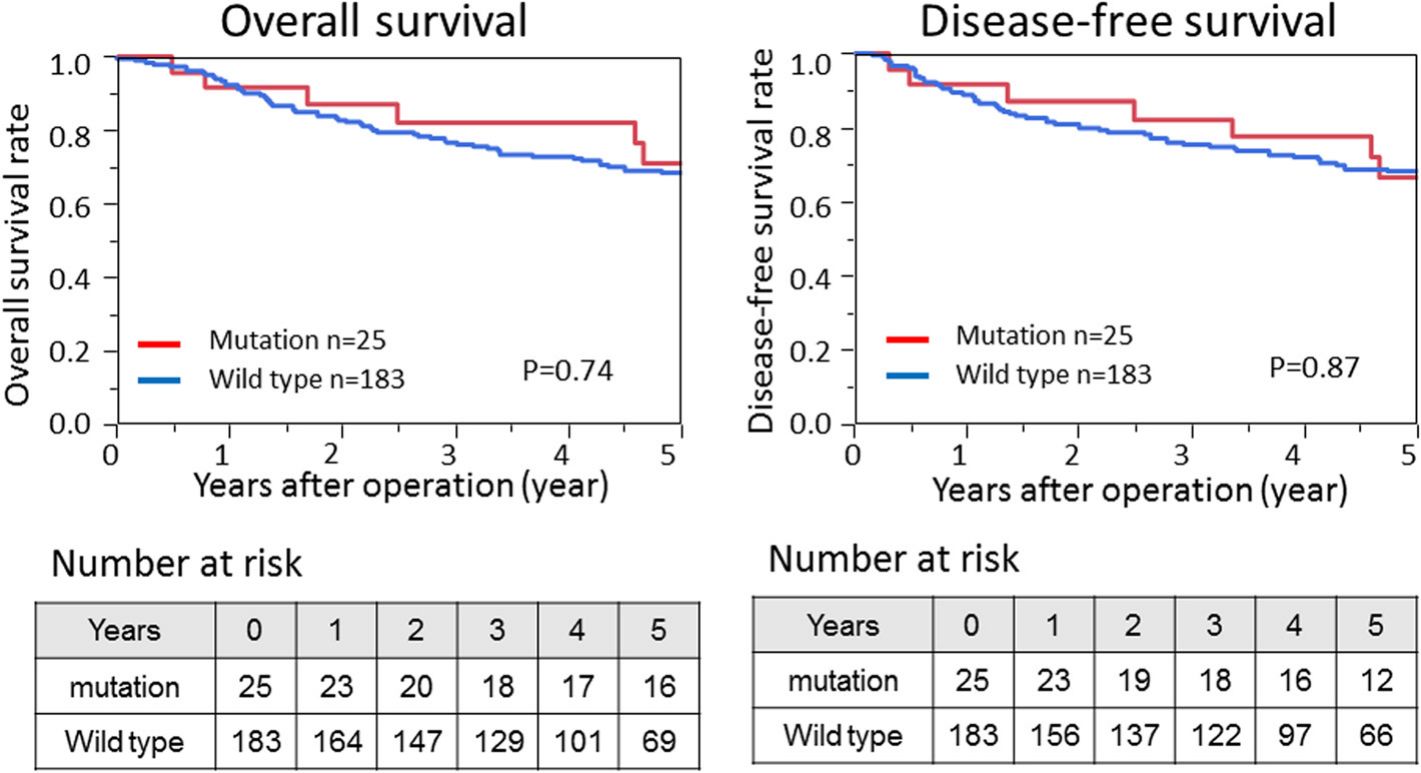
Kaplan–Meier curves showing the relationship of PIK3CA mutational status with overall survival (*left*) and disease-free survival (*right*) in gastric cancer patients

We assessed the influence of PIK3CA mutations on clinical outcome in patients with curatively resected gastric cancer. During follow-up of the 208 patients, 32 patients experienced gastric cancer recurrence and 69 died. The median follow-up time for censored patients was 5.0 years. Kaplan–Meier analysis showed no significant differences in disease-free survival (log rank *P* = 0.84) and overall survival (log rank *P* = 0.74) between patients with PIK3CA mutations and those with PIK3CA wild type (Fig. 2).

### Interaction between PIK3CA mutations and other variables in survival analyses

**Fig. 3:**
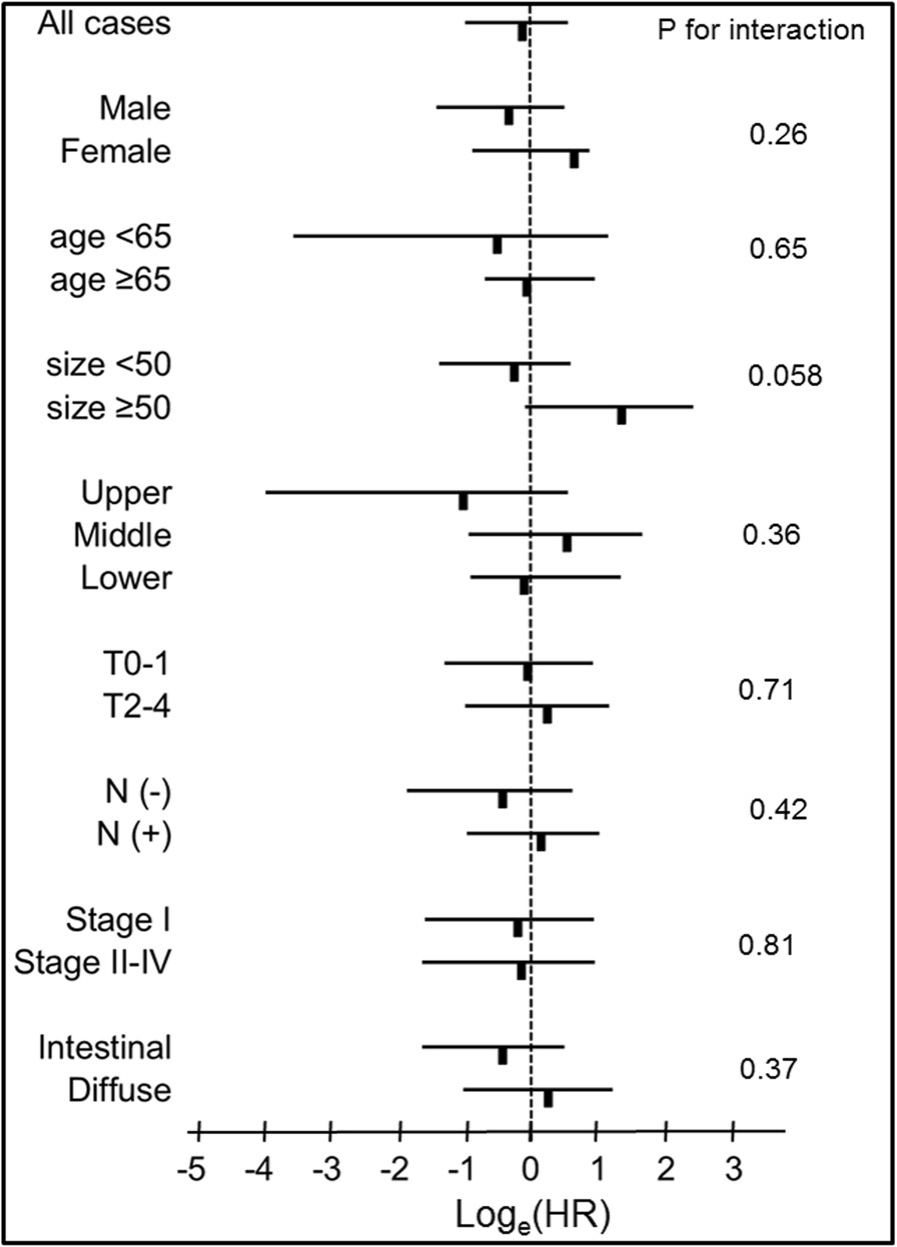
PIK3CA mutations and overall survival in various strata. The log_e_ (adjusted HRs) plot of overall survival in the PIK3CA mutation vs. PIK3CA wild-type group. The 95 % CI is also shown

We also examined whether the influence of PIK3CA mutations on overall survival was modified by any clinical or pathological variable. All tested variables did not significantly interact with the relationship between PIK3CA mutations and overall survival (p for all interactions >0.05, Fig. 3). Moreover, analysis of various strata showed no significant difference in hazard ratio for overall survival between patients with PIK3CA mutations and those with PIK3CA wild type (Fig. 3).

## Discussion

PIK3CA mutations and subsequent activation of the PI3K/AKT pathway play an essential role in cancer cell signaling pathways, involving growth factors, cytokines, and other cellular stimuli associated with human neoplasms [11, 12, 19, 20]. This study examined the prognostic impact of PIK3CA mutations on survival in patients with gastric cancer. Mutations in PIK3CA exons 9 and 20 were observed in 25 of the 208 (12 %) gastric cancer patients. However, overall survival and disease-free survival were similar in patients with and without PIK3CA mutations, indicating that PIK3CA mutations do not affect mortality after resection of gastric cancer.

PIK3CA exons 9 and 20 mutation accounted for over the 80 % of the mutations [6]. Exon 9 mutation exist in helical domain, and exon 20 mutation exist in kinase domain, leading to the activating of AKT pathway and then stimulating the ability of invasion and migration [13]. Especially, E545K mutation disrupts inhibitory interaction between p110 and SH2 domain in p85 [21]. Importantly, Baba et al. have shown the relationship between tumor phosphorylated AKT expression and PIK3CA exons 9 and 20 mutation in 717 colorectal cancer [22]. The oncogenic role of mutations other than hot spot mutation has not been fully investigated. Interestingly, some tumor samples had multiple mutations on exon 9 and 20. Some group have also detected several different PIK3CA mutations in same sample by using next-generation sequencing and Sanger Sequencing. It is possible that one sample harvest three or two different mutations in the same gene [23, 24]. In addition, PIK3CA mutation can coexist with KRAS, EGFR and BRAF [25]. Thus, PIK3CA mutation has been involved in molecular pathway of cancer.

Other studies have reported PIK3CA mutations in 4–25 % of gastric cancers (Table 2). The largest cohort was assessed by the TOGA study group, finding that the PIK3CA mutation rate was 24 %, or about twice as high as in the current study. However, that study evaluated mutations in all exons. A study in a Japanese population found that the mutation rate in PIK3CA exons 1, 9, and 20, as determined by pyrosequencing, was 8.7 % [26]. Directed sequencing found that the mutation rate in PIK3CA exons 9 and 20 was 7.7–15.9 % [27, 28]. Thus, the PIK3CA mutation rate observed in the current study (12 %) was comparable to rates previously reported. Interestingly, the PIK3CA mutation rate in gastric cancer is less than that in colon and breast cancers [29, 30].

**Table 2:** Previous studies of PIK3CA mutations in gastric cancer

Sample number	Mutation rate	Exons evaluated	Method	References
163	5.5 %	9 and 20	Direct sequencing	[36]
263	16 %	9 and 20	Direct sequencing	[27]
12	25 %	18 and 20	Sanger method	[6]
47	11 %	9 and 20	ABI prism 377 automated sequencer	[37]
94	4.3 %	9 and 20	Direct sequencing	[38]
185	6.5 %	9, 18 and 20	PCR – SSCP	[39]
34	6.0 %	9 and 20	High-Resolution	[40]
			Melt Analysis	
295	24 %	All exon	Mut Sig CV	[41]
140	4.0 %	9 and 20	ABI PRISM 3100	[42]
			Genetic Analyzer	
231	8.7 %	1, 9 and 20	Pyrosequencing	[26]
104	7.7 %	9 and 20	Direct sequencing	[28]

Previous studies evaluating the association between PIK3CA mutations and prognosis in human cancers have yielded variable results (Table 3). PIK3CA mutations have been associated with a better prognosis in patients with breast and esophageal cancers, but with a poorer prognosis in patients with colon, rectum, and endometrial cancers [31–35]. These differences may be due to differences in tumor histology. Discovering the mechanisms of this discrepancy is imperative for future projects.

**Table 3:** Studies on prognostic significance of PIK3CA mutations in several types of cancers

Cancer type	Sample number	Mutation rate (%)	Mutation effect	Prognosis	References
Colon cancer	1212	16	Unfavorable	Adjusted CS HR	[31]
				3.51 (1.28–9.62)	
	743	14.5	Unfavorable	Adjusted OS HR	[25]
				3.30 (1.46–7.45)	
	450	18	Unfavorable	Adjusted CS HR	[29]
				2.03 (1.15–3.57)	
Rectal cancer	240	7.9	Unfavorable	Adjusted RS HR	[32]
				3.4 (1.2–9.2)	
Endometrial cancer	1063	16.2	Unfavorable	Not-adjusted RS HR	[33]
				2.18 (1.09–4.39)	
Breast cancer	687	25.3	favorable	Not-adjusted CS HR	[34]
				0.88 (0.58–1.34)	
	439	32.5	Not significant	Adjusted CS HR	[30]
				0.7 (0.4–1.2)	
	188	28.7	favorable	Adjusted RS HR	[35]
				0.42 (0.20–0.92)	
Esophageal cancer	406	7.4	Not significant	Adjusted OS HR	[43]
				1.072 (0.79–1.44)	
	219	21	favorable	Adjusted OS HR	[14]
				0.35 (0.10–0.90)	
Lung cancer	1117	3.4	Not significant	OS log-lank	[44]
				*P* = 0.442	
	235	3.4	Not significant	OS log-lank	[45]
				*P* = 0.15	
Gastric cancer	263	15.9	Not significant	Adjusted OS HR	[27]
				1.1 (0.7–1.7)	
	231	8.7	Not significant	Not-adjusted OS HR	[26]
				1.37 (0.68–3.26)	
	104	7.7	Not significant	OS log-lank	[28]
				*P* = 0.96	
	208	12	Not significant	Not-adjusted OS HR	This study
				0.73 (0.58–2.57)	

*HR* hasard ratio, *CS* cancer specific survival, *RS* recurrence-free survival rate, *OS* overall survival rate

The current study found that PIK3CA mutations were not associated with survival in patients with gastric cancer, in agreement with three previous studies [26–28]. Because of their relatively large sample sizes, including this study that included samples from over 200 samples, these findings, taken together, confirm the lack of relationship between PIK3CA mutations and prognosis in gastric cancer patients. These findings, however, require confirmation by large independent cohort studies.

## Conclusions

In conclusion, mutations in PIK3CA exons 9 and 20 were observed in 25 of the 208 (12 %) gastric cancer patients by pyrosequencing assays. We found that PIK3CA mutations did not correlate with prognosis in patients with gastric cancer, providing additional evidence for the lack of relationship between the two.

## Abbreviations

LINE-1, long interspersed nucleotide element-1; PI3K, phosphoinositide 3-kinase; PIK3CA, phosphatidylinositol-4,5-bisphosphate 3-kinase, catalytic subunit alpha
